# Effects of the Dietary Inclusion of Partially Defatted Black Soldier Fly (*Hermetia illucens*) Meal on the Blood Chemistry and Tissue (Spleen, Liver, Thymus, and Bursa of Fabricius) Histology of Muscovy Ducks (*Cairina moschata domestica*)

**DOI:** 10.3390/ani9060307

**Published:** 2019-05-31

**Authors:** Marta Gariglio, Sihem Dabbou, Manuela Crispo, Ilaria Biasato, Francesco Gai, Laura Gasco, Francesco Piacente, Patrizio Odetti, Stefania Bergagna, Iveta Plachà, Emanuela Valle, Elena Colombino, Maria Teresa Capucchio, Achille Schiavone

**Affiliations:** 1 Department of Veterinary Science, University of Turin, Largo Paolo Braccini 2, 10095 Grugliasco (TO), Italy; marta.gariglio@unito.it (M.G.); sihem.dabbou@unito.it (S.D.); manuela.crispo@unito.it (M.C.); emanuela.valle@unito.it (E.V.); elena.colombino@edu.unito.it (E.C.); mariateresa.capucchio@unito.it (M.T.C.); achille.schiavone@unito.it (A.S.); 2 Department of Agricultural, Forest and Food Sciences, University of Turin, Largo Paolo Braccini 2, 10095 Grugliasco (TO), Italy; ilaria.biasato@unito.it (I.B.); laura.gasco@unito.it (L.G.); 3 Institute of Science of Food Production, National Research Council, Largo Paolo Braccini 2, 10095 Grugliasco (TO), Italy; 4 Department of Internal Medicine and Medical Specialties, University of Genoa, Viale Benedetto XV 6, 16132 Genoa, Italy; francesco.piacente@unige.it (F.P.); odetti@unige.it (P.O.); 5 IRCCS Polyclinic Hospital San Martino, Largo Rosanna Benzi 10, 16132 Genoa, Italy; 6 Veterinary Medical Research Institute for Piemonte, Liguria and Valle d’Aosta, Via Bologna 148, 10154 Turin, Italy; stefania.bergagna@izsto.it; 7 Institute of Animal Physiology, Centre of Biosciences, Slovak Academy of Sciences, Soltesovej 4–6, 040 01 Kosice, Slovak Republic; placha@saske.sk

**Keywords:** poultry, *Hermetia illucens*, insect meal, blood traits, birds, antioxidant, histology features

## Abstract

**Simple Summary:**

Insects represent a promising feed ingredient for poultry diets, as an alternative to conventional feedstuffs. Black soldier fly (BSF; *Hermetia illucens*) larvae are processed to obtain two main products: the protein and fat fractions. The possible utilization of BSF defatted meal in Muscovy duck (*Cairina moschata domestica*) diets has been poorly investigated. However, its effect on *in vivo* and *post-mortem* traits, which are extremely important for animal welfare, has not yet been investigated. Therefore, the present study has evaluated the effect of 0%, 3%, 6%, and 9% dietary BSF meal replacement on the *in vivo* haematological parameters and on the *post-mortem* organ traits. Overall, the obtained results are encouraging as increasing dietary BSF meal did not impair the growth performance or the haematological traits. Furthermore, both the liver and renal function were unaffected or even improved. The antioxidant picture appeared improved and the histological traits were not influenced by the dietary inclusion of BSF meal. From a productive and biological point of view, the dietary replacement up to 9% of BSF meal in Muscovy duck diet is feasible and BSF meal could represent a promising feed ingredient.

**Abstract:**

The present study has evaluated the effects of dietary partially-defatted black soldier fly (BSF; *Hermetia illucens* L.) larva meal on the blood parameters, antioxidant status, and histological features of the organs of broiler ducks. A total of 192 female 3-days of age Muscovy ducklings (*Cairina moschata domestica*) were divided into four dietary treatments (0%, 3%, 6%, and 9% BSF meal inclusion; 6 pens/treatment, 8 birds/pen). A total of 12 ducks/treatment (2 birds/pen) were slaughtered at 51 days of age and blood samples were collected to evaluate the haematological traits, serum protein, lipid and minerals, liver and renal function serum enzymes, plasma oxidative enzymes, and metabolites. Liver, spleen, thymus, and bursa of Fabricius samples were submitted to histopathological investigations. Between the serum and plasma traits, triglycerides, cholesterol, creatinine, alkaline phosphatase, magnesium, malondialdehyde, and nitrotyrosine showed a linear decrease for increasing amounts of dietary BSF meal (*p* <0.01); in contrast, the serum iron concentration showed a linear increase (*p* <0.01). Moreover, the histopathological findings were not significantly affected by the dietary BSF larva meal inclusion. The results showed that the inclusion of up to 9% BSF larva meal represents a promising feed ingredient for Muscovy duck nutrition, and improved blood traits were observed.

## 1. Introduction

In recent years, researchers have paid a great deal of attention to the use of insect-derived products in poultry [1] and in the nutrition of other monogastric animals, as recently shown in reviews by Sogari et al. [2] and Gasco et al. [3]. The main advantage is the low environmental impact of insects, compared to conventional vegetable protein sources, as they require less soil and water, and lead to lower greenhouse gas and ammonia emissions. Indeed, it has been estimated that 1 ha of land can produce less than 1 ton of soybean protein per year compared to 150 tons of insect protein on the same surface [4]. Similarly, a notable contribution is also given by the limited space requirements of insects, together with their ability to grow on organic wastes, their efficient feed conversion ratio, and their high fecundity [5]. Some researchers have reported that the nutritional value of insects is adequate to support poultry growth, nutrient digestibility, and health [6,7,8,9]. However, in other cases, conflicting results have been obtained [10], thus making a thorough study of adequate dietary inclusion levels necessary.

Among the various insect species, black soldier fly (BSF, *Hermetia illucens*) larvae have shown a nutritional composition that is suitable for poultry diets, as it has a high crude protein (CP) content (ranging between 35–57% on a dry matter basis, DM) with a high biological value and an extremely variable ether extract (EE) content (15–49% DM) [11]. The fatty acid composition of BSF larvae depends on the fatty acid composition of the rearing substrate but, generally, the larvae appear rich in lauric acid (20–40% of total lipids), palmitic acid (11–16% of total lipids), and oleic acid (12–32% of total lipids). BSF larvae are also rich in minerals, particularly Ca (5–8% DM) and P (0.6–1.5% DM) [11]. BSF larvae also contain chitin, the main constituent of the exoskeleton of arthropods. The chitin content fluctuates during the life cycle of insects, according to their life stage, but the method used to assess it can lead to dramatic differences in the measurements [12]. The chitin content in BSF larvae ranges from 8.7% [13] to 5.9% [14]. It has been reported that chitin has antioxidant and hypocholesterolemic properties for both humans and animals and appears to have a positive effect on the immune system of poultry, as it exhibits prebiotic properties in the large intestine and appears to exhibit a bacteriostatic effect on Gram-negative bacteria [15,16,17,18]. 

Although the *in vivo* antioxidant effect of chitin has not been investigated in great depth in poultry, it could provide important information about animal welfare. Indeed, the presence of free radicals, especially reactive oxygen species, is associated with several negative biological effects, including deterioration of the DNA, the oxidation of proteins and lipids, and the development of inflammatory disorders [15,19,20]. On the other hand, chitin hypocholesterolemic properties have been mentioned in many studies on broilers and laying hens [16,20,21]. This property could result from the positive charge of this polysaccharide, which binds negatively-charged bile acids and free fatty acids [22]. Finally, the proper functioning and health of the gastrointestinal tract are crucial for ensuring an adequate growth performance in farm animals [23,24]. These aspects are particularly relevant in the poultry industry, where selected birds display an elevated growth potential.

In spite of these interesting biological effects, the use of BSF defatted meal in Muscovy duck (*Cairina moschata domestica*) diets has been poorly investigated so far and, to the best of the authors’ knowledge, there is only one paper that shows encouraging results, in terms of growth performance, diet digestibility, and intestinal morphology in ducks [9], while another paper has assessed the *in vitro* digestibility of different insect meals [25].

Considering this background, the present study has been aimed at investigating the effects of dietary BSF larva meal inclusion on the blood parameters and histological traits of female Muscovy ducks, in order to provide a picture of the animal welfare of Muscovy ducks based on a multidisciplinary approach involving both *in vivo* and *post-mortem* parameters.

## 2. Materials and Methods

### 2.1. Birds and Experimental Design

The present trial was performed at the poultry facility of the University of Turin (Italy). The experimental protocol (prot. no. 380576, 4th December 2017) was approved by the Bioethical Committee of the University of Turin (Italy).

The experimental design of the present study is reported in Gariglio et al. [9]. Briefly, a total of 192 female 3-days of age Muscovy ducklings (Canedins R71 L White, Grimaud Freres Selection, France) were divided into four groups, assigned to four different dietary treatments (6 replicates/treatment and 8 birds/replicate) and raised from 3 to 50 days of age. BSF larva meal was included as a substitute for corn gluten meal (substitution 1:1) at increasing levels (0%, 3%, 6%, and 9%; BSF-0, BSF-3, BSF-6, and BSF-9, respectively) in isonitrogenous and isoenergetic diets formulated for three feeding phases: starter (3–17 d), grower (18–38 d), and finisher (39–50 d). In order to evaluate the effects of dietary BSF larva meal inclusion, all the other ingredients were kept constant, with the exception of the synthetic essential amino acids (DL-methionine and L-lysine), as reported in Table 1. The apparent metabolizable energy (AMEn) of the BSF larva meal has previously been assessed for broiler chickens [26] and was used to formulate the diets of this experiment. The diets were formulated to meet or exceed the nutritional requirements of female Muscovy ducks, as reported by Pingel et al. [27].

### 2.2. Chemical Analysis of the BSF Meal and Experimental Diets

Samples of the experimental diets and BSF larva meal were analyzed for DM (AOAC, #934.01), ash (AOAC, #942.05), CP (AOAC, #984.13), EE (AOAC, #2003.05), neutral detergent fiber (NDF) (AOAC, #2002.04), and acid detergent fiber (ADF) (AOAC, #973.18) [28,29]. The method of Finke et al. [30] was used for the determination of the chitin content of the BSF larva meal using ADF adjusted for its nitrogen content. The chemical composition of the experimental diets is reported in Table 1. Moreover, the chemical composition of the BSF larva meal (on a DM basis) was as follows: 924.1 g/kg DM, 567.1 g/kg CP, 107.0 g/kg EE, 163.8 g/kg ash, and 64.3 g/kg chitin.

### 2.3. Growth Performance

The growth performance parameters were evaluated, as previously reported by Gariglio et al. [9]. The live weight (LW) of the birds was assessed at the beginning and at the end of the trial (at 3 and 50 days of age, respectively), and the average daily gain (ADG), the daily feed intake (DFI), and the feed conversion ratio (FCR) were calculated for the whole experimental period (3–50 days of age). The mortality and health status of birds were monitored on a daily basis.

### 2.4. Slaughtering Procedures and Sampling

At 50 days of age, 12 ducks per diet (two birds per pen) were selected on the basis of the average LW and identified through a shank ring. Subsequently, after a feed withdrawal period of 12 hours (at 51 days of age), the selected ducks were transferred to a commercial processing plant and slaughtered according to the standard EU regulations.

At slaughtering, blood samples were collected in EDTA tubes and serum-separating tubes, further details of which are provided in Section 2.5.

Immediately after the completion of the slaughtering phase, spleen, liver, thymus, and bursa of Fabricius samples were collected and fixed in a 10% buffered formalin solution for histochemical staining, further details of which are provided in Section 2.6.

### 2.5. Blood Analysis

Blood samples were collected, at slaughtering, from the jugular vein of twelve birds (two animals per pen) per feeding group. An aliquot of 2.5 mL was placed in an EDTA tube and 2.5 mL in a serum-separating tube. A blood smear was prepared from a droplet without any anticoagulant. The total red (erythrocytes) and white (leukocytes) cell counts were determined in an improved Neubauer haemocytometer after mixing with a Natt-Herrick solution in a 1 to 200 ratio, as reported by Natt and Herrick [31]. The blood smears were stained with May-Grünwald and Giemsa–Romanowski stains. One hundred white blood cells were evaluated per smear to determine the heterophils to lymphocytes (H/L) ratio, while the number of blood cell types was determined according to Campbell [32].

The serum-separating tubes were left in a standing position, at room temperature, for approximately two hours, until the formation of a blood clot. Subsequently, the tubes were centrifugated at 700× *g* for 15 minutes and the obtained serum was immediately frozen at −80 °C. The total protein was quantified using the “biuret method” (Bio Group Medical System kit; Bio Group Medical System, Talamello (RN), Italy); the electrophoretic pattern of the serum was assessed using a semi-automated agarose gel electrophoresis system (Sebia Hydrasys®, Norcross, GA, USA). The alanino-aminotransferase (ALT), aspartate-aminotransferase (AST), gamma glutamyl transferase (GGT), alkaline phosphatase (ALP), triglycerides, cholesterol, Ca, P, Mg, Fe, uric acid, and creatinine serum concentrations were measured using enzymatic methods in a clinical chemistry analyzer (Screen Master Touch, Hospitex diagnostics Srl., Firenze, Italy), as described by Salamano et al. [33].

In order to obtain plasma, the EDTA tubes were centrifugated at 2000× *g* for 10 minutes to separate the cell fractions, and the supernatants were immediately frozen at −80 °C and then used to determine the antioxidant status and oxidative metabolites. The blood glutathione peroxidase (GPx, EC 1.11.1.9) and total antioxidant status (TAS) activities of the plasma were determined using a Ransel Enzymatic Kit (RS504, Randox Laboratories, Crumlin, UK) and a TAS Colorimetric Kit (NX2332, Randox Laboratories, Crumlin, UK), respectively, according to the manufacturer’s recommendations.

The enzyme immunoassay for the detection and quantification of methylglyoxal (MG) was performed using an OxiSelect^TM^ Methylglyoxal ELISA Kit (STA-811, Cell Biolabs, San Diego, CA, USA), while an OxiSelect^TM^ MDA Adduct Competitive ELISA Kit (STA-832, Cell Biolabs, San Diego, CA, USA) was used for the malondialdehyde (MDA) quantification. Both tests were performed on plasma samples. The 3-nitrotyrosine plasma concentration was measured by an enzyme-linked immunosorbent assay (ELISA) using an OxiSelect^TM^ Nitrotyrosine ELISA Kit (STA-305, Cell Biolabs, San Diego, CA, USA). All the tests were performed according to the manufacturer’s instructions.

All the analyses were performed in duplicate.

### 2.6. Histological Investigations

The slaughtered birds (n = 12 per experimental diet, two birds per pen) were submitted to an anatomopathological examination. Spleen (entire organ), liver (left lobe), thymus (left side lobes), and bursa of Fabricius (entire organ) samples were collected (0.5–1.5 g/organ) and fixed in a 10% buffered formalin solution, embedded in paraffin wax blocks, sectioned at a thickness of 5 μm, mounted onto glass slides, and stained with Haematoxylin & Eosin for the histopathological examination [34]. The following histopathological alterations were evaluated: white pulp hyperplasia and depletion in the spleen, cortical depletion in the thymus, follicular depletion and intrafollicular cysts in the bursa of Fabricius, and hepatocyte degeneration and lymphoid tissue activation in the liver [6].The observed histopathological alterations were evaluated using a semiquantitative scoring system as follows: absent (score = 0), mild (score = 1), moderate (score = 2), and severe (score = 3). In order to investigate the accumulation of lipids and polysaccharides in the liver, tissue samples of these organs were also stained with Sudan Black and Periodic acid-Schiff (PAS), respectively. The lipid and polysaccharide staining intensity was scored semi-quantitatively as follows: grade 0 for an absence of staining, grade 1 for mild staining, grade 2 for moderate staining, and grade 3 for marked staining. All the slides were blindly evaluated by three different observers and the discordant cases were reviewed using a multi-head microscope until a unanimous consensus had been reached.

### 2.7. Statistical Analysis

The statistical analysis was performed using the SPSS software package (version 21 for Windows, SPSS Inc., Chicago, IL, USA). The mortality rate was analyzed by means of a Chi-square test, using the BSF0 group as reference. Shapiro-Wilk’s test was used to establish the normality or non-normality of the distributions. The assumption of equal variances was assessed by means of Levene’s homogeneity of variance test. The birds’ pen was identified as the experimental unit to evaluate the growth performance, while the blood traits and histological features were evaluated individually for each duck. The collected data were tested using one-way ANOVA. Polynomial contrasts were used to test the linear and quadratic responses to increased levels of BSF inclusion in the diet. Histopathological scores were analyzed by means of the Kruskal-Wallis test (post-hoc test: Dunn’s Multiple Comparison test). Differences between treatments were considered statistically significant when the *p* values ≤ 0.05.

## 3. Results

### 3.1. Growth Performance

The growth performance of the broiler ducks, as reported in detail by Gariglio et al. [9], is summarized in Table 2. Overall, LW, ADG, DFI, and FCR were not influenced by the dietary treatments (*p* >0.05). The cumulative mortality rates were 4.16 % (for BSF-0) and 2.08% (for BSF-3, BSF-6, and BSF-9) and no statistical effect of dietary treatment was found for this parameter (*p* >0.05).

### 3.2. Blood Traits

As reported in Table 3, the haematological traits of the Muscovy ducks fed up to 9% dietary BSF larva meal were not influenced by the dietary treatments. Serum protein was similar between groups, while the triglycerides and cholesterol levels displayed a linear decrease (*p* <0.05), as a result of increasing levels of dietary BSF larva meal (30.23% and 23.86% lower in BSF9 than BSF0, respectively; Table 3).

Table 4 reports the results for the serum minerals and for the liver and renal function parameters. Ca and P were unaffected by the dietary treatments, while a linear response was observed for the Mg and Fe concentrations. In particular, Mg showed a linear decrease as a result of the increasing dietary inclusion levels of BSF larva meal, while Fe showed a linear increase, with a maximum for the BSF-9 group (*p* <0.01).

The parameters associated with the liver functions, including AST, ALT, and GGT, were unaffected for all the groups, while ALP showed a linear decrease (*p* <0.01).

The renal function was partially influenced by the dietary inclusion of BSF larva meal. No changes in the levels of uric acid were observed. However, a linear decrease in the creatinine values (*p* <0.05) was identified.

As reported in Table 5, the antioxidant enzymes and oxidative metabolites picture was partially influenced by the dietary treatments. GPx, TAS, and MG remained constant between groups, while MDA and nitrotyrosine showed a linear decrease with increasing dietary levels of BSF (*p* <0.001).

### 3.3. Histological Investigations

No macroscopic lesions were detected during the anatomopathological examination in any of the slaughtered birds. Histopathological changes were identified in all the examined organs collected from the birds belonging to all the dietary treatments. Regardless of the dietary treatment, the spleen showed mild, multifocal white pulp hyperplasia (range score: 0.20–0.42), while mild, multifocal cortical depletion was observed in the thymus (range score: 0.00–0.18). Furthermore, mild to moderate, multifocal to diffuse follicular depletion was detected in the bursa of Fabricius (range score: 0.00–0.36), whereas the liver showed mild to severe, multifocal to diffuse steatosis (mild to marked SB staining intensity) or vacuolar degeneration of the hepatocytes (range score: 0.77–1.88). No polysaccharide accumulation was observed in the hepatocytes (absence of PAS positivity). The dietary BSF larva meal inclusion did not affect the severity of the observed histopathological changes (*p* >0.05, Table 6).

## 4. Discussion

### 4.1. Growth Performance

The first part of the study evaluated the use of BSF larva meal in Muscovy ducks from a nutritional point of view [9], and this was followed by the investigation of the blood chemistry and tissue histology reported in this paper. The percentages of BSF larva meal included in the feeds used in the study appear compatible with the digestive physiology of the Muscovy duck, since no detrimental effects on growth, digestibility, or intestinal morphology were noted [9]. A low mortality rate and a lack of clinical signs indicates that the inclusion of low levels of dietary BSF larva meal does not have any detrimental effects on the health of the birds. This is further supported by the results of the blood chemistry investigation and tissue histological findings discussed below.

### 4.2. Blood Traits

The blood parameters considered in this study provide an overview of the health status and the welfare of birds. The unaffected haematological picture, as well as the H/L ratio (which is related to the presence of stressful conditions [35]), support the lack of stress resulting from the use of BSF larva meal in Muscovy duck diets. These results are in line with what has been reported for broiler chickens [10], laying hens [16,21], and Barbary partridges [7]. In addition, the plasma protein levels were similar between groups, thus suggesting a similar dietary protein bioavailability. However, the blood triglycerides and cholesterol levels appeared reduced as the dietary inclusion levels of BSF larva meal were increased, as has also been described for laying hens [16,21]. A possible explanation of this effect could be due to the chelating effect of chitin in the BSF larva meal [22,23,36].

In our study, Ca and P were not influenced by the dietary treatments. However, Mg showed a linear decrease and Fe a linear increase. The results obtained in the present study can be contextualized within the wide variability of the findings reported in other studies [7,10,16,21].

The functioning of the organs appeared preserved or improved in all the experimental groups, as shown by the liver and renal serum enzyme levels. Indeed, the liver enzymes, as confirmed by the ALT, AST, and GGT concentrations, remained constant between treatments. In addition, ALP was found to be lower in the insect-fed groups than the BSF-0 (8.61% lower in BSF-9 than BSF-0), thus demonstrating the absence of adverse effects of dietary BSF larva meal inclusion on the liver function of Muscovy ducks, as previously demonstrated for broiler chickens [10]. On the other hand, the unchanged uric acid levels and the linear decrease in the creatinine levels showed a partially improved renal function. This result is in line with what Marono et al. [16] observed for laying hens. Although chitin is not associated with renal functioning, some papers have reported that chitosan, produced by chitin deacetylation, was able to improve the renal function of rats and humans, and that they showed decreased serum creatinine levels [37,38,39].

In the present paper, the blood and plasma antioxidant enzymes (GPx and TAS) were not affected by the BSF larva meal inclusion levels. On the other hand, the plasma oxidative metabolites were affected by the dietary treatments, and showed decreased values of MDA and nitrotyrosine as a result of the dietary inclusion levels of BSF larva meal. The MDA is the end product of lipid breakdown, as a result of oxidative stress [40]. Furthermore, the lipid peroxidation process involves the oxidation of tyrosine with the formation of nitrotyrosine, which promotes the oxidative tissue damage [41]. Increased levels of 3-nitrotyrosine have been correlated with high levels of other indices of oxidative stress markers associated with apoptotic cell death [42,43]. Although nitrotyrosine is a commonly evaluated parameter in humans and rats [44,45], only a few studies have conducted an assessment of nitrotyrosine as an oxidative stress marker in poultry [46,47]. Although the positive effects of dietary antioxidants have been investigated extensively in Muscovy ducks [48,49,50], the antioxidant effect of chitin has not yet been elucidated. The potential free radical scavenging effect of chitin may play an important role in animal health by preserving cellular integrity and the normal biological functions.

### 4.3. Histological Investigations

The dietary BSF larva meal inclusion did not significantly influence either the development or severity of the histopathological alterations detected in the ducks of the current research. This is in agreement with Biasato et al. [6,51,52] and Dabbou et al. [10], who did not find any significant alterations in broiler chickens fed diets with yellow mealworm (*Tenebrio molitor,* TM) or BSF inclusions, respectively. Regardless of the dietary treatment, the ducks of the four experimental groups showed more severe hepatic lesions than those recorded in broiler chickens fed TM meal [6,51,52] or BSF meal [10]. This difference can be explained by considering the genetic predisposition of ducks to developing hepatic steatosis under intensive farming conditions [53,54]. Furthermore, Chartrin et al. [55] and Hérault et al. [56] reported that Muscovy ducks showed a greater tendency to accumulate lipids in the liver than Pekin ducks. Lipid mobilization from the liver to the peripheral tissues is generally related to the availability of transport lipoproteins [57]. On the basis of these findings, it is possible to state that the BSF larva meal showed no negative effects on animal health.

## 5. Conclusions

Our study provides original and novel information about the use of BSF larva meal in the nutrition of Muscovy ducks. Increasing the dietary levels of BSF larva meal did not impair the growth performance or haematological traits of the birds. Furthermore, both the liver and renal functions were unaffected or even improved. According to the literature, the serum minerals have provided conflicting results, thus underlying the need for additional studies on this topic. The antioxidant picture was improved, and the histological traits were not influenced by the addition of the dietary BSF larva meal. Moreover, some metabolites (MG and nitrotyrosine) were evaluated for the first time in Muscovy ducks. From a productive and biological point of view, the BSF larva meal, at inclusion levels of up to 9%, represents a promising feed ingredient for the nutrition of Muscovy ducks.

## Figures and Tables

**Table 1:** Ingredients (g/kg as fed) and nutrient composition (%) of the experimental diets.

Ingredients	Starter period (3 to 17 d) ^1^				Grower period (18 to 38 d) ^1^				Finisher period (39 to 50 d) ^1^			
	BSF-0	BSF-3	BSF-6	BSF-9	BSF-0	BSF-3	BSF-6	BSF-9	BSF-0	BSF-3	BSF-6	BSF-9
Corn meal	600.0	600.0	600.0	600.0	638.0	638.0	638.0	638.0	670.0	670.0	670.0	670.0
Soybean meal	212.0	212.0	212.0	212.0	160.0	160.0	160.0	160.0	100.0	100.0	100.0	100.0
BSF larva meal	0.0	30.0	60.0	90.0	0.0	30.0	60.0	90.0	0.0	30.0	60.0	90.0
Bran	42.5	42.5	42.5	42.5	36.3	36.3	36.3	36.3	66.2	66.2	66.2	66.2
Corn gluten meal	90.0	60.0	30.0	0.0	90.0	60.0	30.0	0.0	90.0	60.0	30.0	0.0
Soybean oil	16.5	16.5	16.5	16.5	28.5	28.5	28.5	28.5	34.5	34.5	34.5	34.5
Dicalcium phosphate	10.0	10.0	10.0	10.0	13.0	13.0	13.0	13.0	4.0	4.0	4.0	4.0
Calcium carbonate	8.0	8.0	8.0	8.0	14.0	14.0	14.0	14.0	17.4	17.4	17.4	17.4
Sodium chloride	2.5	2.5	2.5	2.5	2.5	2.5	2.5	2.5	2.5	2.5	2.5	2.5
Sodium bicarbonate	2.0	2.0	2.0	2.0	2.0	2.0	2.0	2.0	2.0	2.0	2.0	2.0
DL-methionine	2.5	2.5	2.6	2.8	1.7	1.8	1.9	2.2	0.3	0.4	0.5	0.8
L-lysine	3.9	3.9	3.8	3.6	3.9	3.8	3.7	3.4	3.0	2.9	2.8	2.5
MinVit premix^2^	5.0	5.0	5.0	5.0	5.0	5.0	5.0	5.0	5.0	5.0	5.0	5.0
Choline chloride	0.1	0.1	0.1	0.1	0.1	0.1	0.1	0.1	0.1	0.1	0.1	0.1
Optifos 250 bro^3^	1.0	1.0	1.0	1.0	1.0	1.0	1.0	1.0	1.0	1.0	1.0	1.0
Avizyme 1500 x^4^	1.0	1.0	1.0	1.0	1.0	1.0	1.0	1.0	1.0	1.0	1.0	1.0
Titanium dioxide	3.0	3.0	3.0	3.0	3.0	3.0	3.0	3.0	3.0	3.0	3.0	3.0
Total	1000	1000	1000	1000	1000	1000	1000	1000	1000	1000	1000	1000
AMEn, MJ/kg	12.12	12.10	12.08	12.07	12.53	12.51	12.49	12.47	12.77	12.75	12.74	12.72
Nutrient composition												
DM	89.3	89.8	90.1	89.7	88.8	89.2	89.1	89.1	88.7	89.0	89.3	88.9
CP, % DM	25.1	24.7	25.2	24.8	23.0	22.5	22.5	22.4	20.2	20.2	20.1	20.1
EE, % DM	4.7	4.8	4.9	5.1	6.2	6.2	6.4	6.6	7.3	7.4	7.6	7.7
NDF, % DM	12.7	12.3	12.7	12.4	12.9	13.1	12.6	12.7	12.7	13.1	12.7	12.9
ADF, % DM	3.4	3.5	3.7	3.3	3.5	3.5	3.7	3.5	3.4	3.5	3.5	3.4
Ash, % DM	5.6	6.0	5.6	5.8	7.8	7.5	7.5	8.0	6.5	6.4	6.9	6.7

Note: BSF: black soldier fly; AMEn: apparent metabolisable energy; DM: dry matter; CP: crude protein; EE: ether extract; NDF: neutral detergent fiber; ADF: apparent digestible fiber. ^1^BSF-0, BSF-3, BSF-6, and BSF-9 = dietary inclusion of BSF larva meal at 0%, 3%, 6%, and 9%, respectively. ^2^Mineral-vitamin premix. Composition per kg of diet: vitamin A (retinyl acetate), 62.5 IU; vitamin D3 (cholecalciferol), 17.5 IU; vitamin E (DL-a-tocopheryl acetate), 200 µg; vitamin K (menadione sodium bisulfite), 10 µg; biotin, 1 µg; thiamine, 10 µg; riboflavin, 30 µg; pantothenate, 76.05 µg; niacin, 200 µg; choline, 3750 µg; pyridoxine, 20 µg; folic acid, 3.75 µg; vitamin B12, 0.15 µg; Mn, 350 µg; Zn, 310.75 µg; Fe, 250 µg; Cu, 350 µg; I, 1.25 µg; Se, 1.25 µg. ^3^Optifos 250 bro: Phytase (EC 3.1.3.26) (250 OTU/kg diet), Huvepharma, Sofia, Bulgaria. ^4^Avizyme 1500 x: Complex of Endo 1-4-Beta- Xilanase (EC 3.2.1.8) (256 U/kg), subtilisine (Ec 3.4.21.62) (2560 U/kg diet) and alpha-amylase (EC3.2.1.1) (1472 U/kg diet), Danisco Animal Nutrition, Marlborough, Wiltshire, UK.

**Table 2:** Growth performance of Muscovy ducks fed increasing BSF meal from 3 to 50 d of age (n = 6 pen; 8 birds/pen).

Items	Age	Dietary Treatments ^1^				SEM	*p*-value	
		BSF-0	BSF-3	BSF-6	BSF-9		Linear	Quadratic
LW, g	3 d	70.7	70.4	72.6	71.5	0.60	0.405	0.733
LW, g	50 d	2540.6	2511.1	2456.1	2554.8	20.13	0.946	0.123
ADG, g/d	3–50 d	52.5	51.9	50.7	52.8	0.43	0.926	0.125
DFI, g/d	3–50 d	120.6	121.3	117.6	121.5	1.20	0.927	0.530
FCR, g/g	3–50 d	2.29	2.34	2.32	2.30	0.019	0.925	0.406

Note: BSF: black soldier fly; SEM: standard error of the mean; LW: live weight; ADG: average daily gain; DFI: daily feed intake; FCR: feed conversion ratio. ^1^BSF-0, BSF-3, BSF-6, and BSF-9 = dietary inclusion of BSF larva meal at 0%, 3%, 6%, and 9%, respectively.

**Table 3:** Haematological traits and the serum protein and lipids of Muscovy ducks fed increasing BSF larva meal from 3 to 50 d of age (n = 12 birds/dietary treatment).

Items	Dietary Treatments ^1^				SEM	*p*-value	
	BSF-0	BSF-3	BSF-6	BSF-9		Linear	Quadratic
Haematological Traits							
Erythrocytes, 10^6^, cell/μL	5.39	4.98	4.98	4.92	0.11	0.160	0.429
Leukocytes, 10^3^, cell/μL	17.58	17.52	18.08	18.03	0.34	0.547	0.991
Heterophils, %	50.83	51.75	46.18	51.16	1.31	0.697	0.443
Lymphocytes, %	47.75	46.17	50.91	46.58	1.36	0.920	0.623
Monocytes, %	0.58	1.00	1.09	0.83	0.14	0.501	0.235
Eosinophils, %	0.33	0.75	1.18	1.17	0.19	0.092	0.576
Basophils, %	0.50	0.33	0.64	0.25	0.12	0.673	0.646
H/L	0.98	0.96	0.96	0.97	0.03	0.997	0.876
Serum proteins and lipids							
Total Protein, g/dl	4.26	4.84	4.99	4.79	0.11	0.086	0.082
Triglycerides, mg/dl	73.27	58.38	55.93	51.12	3.08	0.012	0.395
Cholesterol, mg/dl	90.79	85.25	82.71	69.13	3.13	0.016	0.507

Note: BSF: black soldier fly; SEM: standard error of the mean; H/L: Heterophils to lymphocytes ratio. ^1^BSF-0, BSF-3, BSF-6, and BSF-9 = dietary inclusion of BSF larva meal at 0%, 3%, 6%, and 9%, respectively.

**Table 4:** Serum minerals and the liver and renal functions of Muscovy ducks fed increasing levels of BSF larva meal from 3 to 50 d of age (n = 12 birds/dietary treatment).

Items	Dietary Treatments^1^				SEM	p-value	
	BSF-0	BSF-3	BSF-6	BSF-9		Linear	Quadratic
Minerals							
Ca, mg/dl	10.02	10.59	10.56	9.27	0.37	0.469	0.840
P, mg/dl	3.87	4.35	3.99	3.93	0.16	0.900	0.407
Mg, mg/dl	1.48	1.43	1.32	1.25	0.03	0.002	0.858
Fe, mg/l	310.22	327.33	371.18	406.39	13.80	0.007	0.731
Liver function							
AST, U/l	27.14	27.32	27.08	28.58	0.89	0.622	0.717
ALT, U/l	27.54	30.49	29.48	25.41	1.30	0.530	0.187
GGT, U/l	4.83	4.23	4.82	4.92	0.23	0.685	0.452
ALP, U/l	2003.6	1889.8	1876.4	1831.1	22.59	0.008	0.426
Renal function							
Uric acid, mg/dl	3.98	3.88	3.69	3.31	0.17	0.152	0.679
Creatinine, mg/dl	0.30	0.29	0.29	0.25	0.01	0.022	0.526

Note: BSF: black soldier fly; SEM: standard error of the mean; Ca: calcium; P: phosphorus; Mg: magnesium; Fe: iron; AST: aspartate-aminotransferase; ALT: alanine-aminotransferase; GGT: gamma-glutamyl transferase; ALP: alkaline phosphatase. ^1^BSF-0, BSF-3, BSF-6, and BSF-9 = dietary inclusion of BSF larva meal at 0%, 3%, 6%, and 9%, respectively.

**Table 5:** Antioxidant enzymes and oxidative metabolites of the Muscovy ducks fed increasing levels of BSF larva meal from 3 to 50 d of age (n = 12 birds/dietary treatment).

Items	Dietary treatments ^1^				SEM	*p*-value	
	BSF-0	BSF-3	BSF-6	BSF-9		Linear	Quadratic
GPx, U/g Hb	216.54	240.27	229.74	246.39	7.51	0.250	0.816
TAS, mmol/l	1.48	1.47	1.46	1.43	0.03	0.618	0.870
MG, μg/ml	0.49	0.50	0.47	0.46	0.01	0.307	0.747
MDA, pmol/ml	161.99	169.97	148.73	147.10	2.10	0.000	0.152
Nitrotyrosine, nM	193.10	133.42	122.66	78.67	11.01	0.000	0.677

Note: BSF: black soldier fly; SEM: standard error of the mean; GPx: glutathione peroxidase; Hb: haemoglobin; TAS: total antioxidant status; MG: methylglyoxal; MDA: malondialdehyde. ^1^BSF-0, BSF-3, BSF-6, and BSF-9 = dietary inclusion of BSF larva meal at 0%, 3%, 6%, and 9%, respectively.

**Table 6:** Histopathological scores of the Muscovy ducks fed increasing BSF larva meal from 3 to 50 d of age (n = 12 birds/dietary treatment).

Items	Dietary Treatments^1^				SEM	*p*-value
	BSF-0	BSF-3	BSF-6	BSF-9		
**Spleen**	**0.42**	0.29	0.27	0.20	0.07	0.740
Liver	1.38	1.88	0.77	1.38	0.15	0.096
Thymus	0.10	0.18	0.09	0.00	0.06	0.549
Bursa of Fabricius	0.33	0.25	0.00	0.36	0.07	0.224

Note: BSF: black soldier fly; SEM: standard error of the mean. ^1^BSF-0, BSF-3, BSF-6, and BSF-9 = dietary inclusion of BSF larva meal at 0%, 3%, 6%, and 9%, respectively.
